# Idelalisib: The First-in-Class Phosphatidylinositol 3-Kinase Inhibitor for Relapsed CLL, SLL, and Indolent NHL

**DOI:** 10.6004/jadpro.2014.5.6.6

**Published:** 2014-11-01

**Authors:** Nicholas Forcello, Nidhi Saraiya

**Affiliations:** Hartford Hospital, Hartford, Connecticut

Chronic lymphocytic leukemia (CLL) is a malignancy arising from a monoclonal population of mature B cells (Chiorazzi, Rai, & Ferrarini, 2005). Chronic lymphocytic leukemia is typically considered a disease of the elderly with a median age at diagnosis of approximately 70 years and is the most common adult leukemia in Westernized countries (Smolewski, Witkowska, & Korycka-Wolowiec, 2013). When these cells are predominantly found in the lymph nodes as compared to the bone marrow, the disease is referred to as small lymphocytic lymphoma (SLL; National Comprehensive Cancer Network [NCCN], 2014). As these disorders share some common characteristics with other B-cell neoplasms, they are frequently grouped in the classification known as indolent non-Hodgkin lymphoma (NHL).

While the natural progression of CLL varies according to the genetic and pathophysiologic characteristics of the cancer cells, treatment has traditionally depended more on patient factors than tumor factors. Chemotherapy regimens including both alkylating agents and purine analogs have shown significant efficacy against this disease, despite their inherent toxicities (NCCN, 2014). Since no benefit has been derived from treating early nonsymptomatic disease, treatment is typically deferred until clinical symptoms present. Standard therapy for younger patients without significant comorbidities includes more aggressive regimens like fludarabine, cyclophosphamide, and rituximab, also known as FCR, while elderly patients or those with significant comorbidities receive more easily tolerated regimens like bendamustine and rituximab, also known as BR, or rituximab monotherapy (NCCN, 2014; Byrd et al., 2014).

Although significant gains have been realized through the incorporation of newer less toxic, targeted therapies, there still remains a subset of patients with limited treatment options. These patients include those with unmutated IGHV, 17p deletions, and relapsed or refractory disease who typically respond poorly to current standards of care. With the exception of the anti-CD20 therapies, few targeted mechanisms have been available until recently. Our growing knowledge of B-cell receptor (BCR) signaling, however, has paved the way for many new agents currently under investigation. One of these new therapies is the phosphatidylinositol 3-kinase delta (PI3Kä) inhibitor idelalisib (Zydelig).

## PHOSPHATIDYLINOSITOL 3-KINASE AS AN ATTRACTIVE TARGET

BCR signaling, both antigen-dependent and independent, is critical to CLL survival and results from increased expression of certain survival pathways (Byrd et al., 2014). Many of these survival pathways exist throughout human physiology, and include nuclear factor κB (NF-κB), mitogen-activated protein kinase (MAPK), Bruton’s tyrosine kinase and phosphatidylinositol 3-kinase (PI3K; Byrd et al., 2014; Hoellenriegel et al., 2011; Zhang & Yu, 2010). The PI3K family consists of 3 classes, each including multiple isoforms. While many of these isoforms are ubiquitous, the PI3K class 1 delta isoform (PI3Kä) has been found to be relatively specific to lymphocytic hematopoietic cells (Hoellenriegel et al., 2011).

PI3K produces a lipid product, phosphatidylinositol-3,4,5-triphosphate (PIP3), which serves to activate Akt, also referred to as protein kinase B or PKB (Hoellenriegel et al., 2011; Zhang & Yu, 2010). The tumor suppressor phosphatase and tensin homolog (PTEN) antagonizes this action by hydrolyzing PIP3 to PIP2 (Zhang & Yu, 2010). When PI3K is overexpressed or PTEN is impeded, Akt activation is increased. This then increases activation of its downstream targets, including forkhead box proteins (FOXO), glycogen synthase kinases (GSK), mammalian target of rapamycin (mTOR), and several others, which ultimately serve to drive cellular metabolism and resistance to apoptosis (Hoellenriegel et al., 2011; Zhang & Yu, 2010). This pathway has been cited as one of the most significant drivers of tumor proliferation and progression (Zhang & Yu, 2010). Idelalisib is an oral, selective PI3Kä inhibitor, the first PI3K inhibitor to be approved by the US Food and Drug Administration (FDA) and one of many PI3K inhibitors currently being produced and tested in clinical trials (Zhang & Yu, 2010; Furman et al., 2014).

## IDELALISIB TRIAL RESULTS

Idelalisib, previously known as GS-1101 and CAL-101, in combination with rituximab was investigated in a phase III, multicenter, randomized, double-blind, placebo-controlled trial in 220 CLL patients with relapsed disease (Furman et al., 2014). Eligible patients needed to have disease progression within 24 months of their last treatment, previously received anti-CD20 therapy or ≥ 2 prior cytotoxic therapies and had current contraindications to cytotoxic therapy. All patients received rituximab 375 mg/m² intravenously for the first cycle and at 500 mg/m² intravenously on subsequent cycles. Cycles were every 2 weeks for 5 doses, then monthly for 3 doses. Patients were randomized to receive either idelalisib 150 mg orally twice daily or placebo with a primary endpoint of progression-free survival (PFS). There were no statistically significant differences in baseline characteristics between the groups, with 78% of all patients being ≥ 65 years old, > 80% having unmutated IGHV, > 40% having a 17p deletion, and 85% having a Cumulative Illness Rating Scale (CIRS) score of more than 6.

The 24-week PFS was 93% and 46% for the idelalisib and placebo groups, respectively, which resulted in the trial being stopped early due to treatment efficacy. The median duration of PFS was not reached in the idelalisib group and 5.5 months in the placebo group. The median duration of idelalisib and placebo treatment was 3.8 and 2.9 months, respectively, though 81% of idelalisib patients were continuing treatment at study termination compared to 52% of patients receiving placebo. The most impressive finding was that idelalisib and rituximab treatment had similar efficacy regardless of the presence of 17p deletion or IGHV mutational status. A secondary endpoint of overall survival was 92% vs. 80% in favor of idelalisib.

Idelalisib has also been investigated in a phase II, single-arm, multicenter, open-label study in 125 patients with relapsed indolent B-cell NHL refractory to both rituximab and an alkylating agent (Gopal et al., 2014). Idelalisib was administered at 150 mg orally twice daily until disease progression, unacceptable toxicity, or death, with the primary endpoint investigating overall response rate. The median age of the patients in this study was 64 years, 80% had either follicular lymphoma or SLL, and 79% had either intermediate or high-risk disease as assessed by the Follicular Lymphoma International Prognostic Index (FLIPI). The response rate was 57% as evaluated by the independent review committee. No significant differences in response rates were found between subgroups. The median time to response was 1.9 months, the median duration of response was 12.5 months, and the rate of PFS at 48 weeks was 47%.

## DOSING AND ADMINISTRATION

Idelalisib is available in 100- and 150-mg tablets. The recommended starting dose is 150 mg given orally twice daily without regard to meals. Tablets should be swallowed whole. Refer to Table 1 for recommendations on dose adjustments. As the median duration of treatment was 6.6 months in the phase II trial and 3.8 months in the phase III trial, the long-term safety profile and optimal duration of therapy have not been defined. Treatment should be continued until no longer tolerated or disease progression (Gilead, 2014).

**Table 1:**
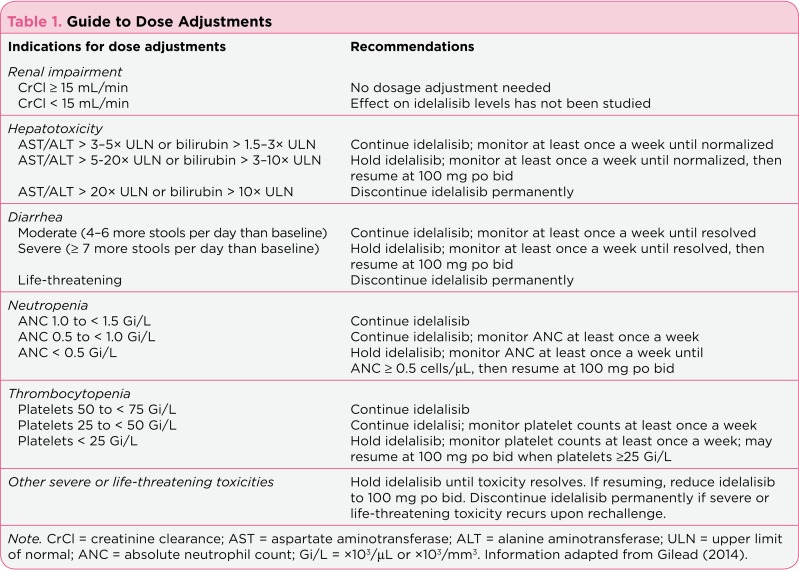
Guide to Dose Adjustments

Idelalisib is mainly metabolized by CYP3A enzymes with minor metabolism via UGT1A4. Avoid concomitant use with strong CYP3A inducers (e.g., carbamazepine, rifampin) and monitor closely for increased risk of toxicities if used with strong CYP3A inhibitors (e.g. clarithromycin, itraconazole, ritonavir). Caution is advised when coadministering CYP3A substrates, as idelalisib is a strong CYP3A inhibitor and dose adjustments or changes in therapy may be required (Gilead, 2014).

## ADVERSE EFFECTS

In the phase II trial, the five most common adverse events reported were diarrhea, nausea, fatigue, cough, and pyrexia. Grade 3 or 4 diarrhea or colitis occurred in 16% of patients after a median duration of 6 months. Idelalisib-related diarrhea appears to be late-onset and may not have been fully elucidated in the available trials. Other grade 3 or 4 events included neutropenia, elevated serum aminotransferases, thrombocytopenia, anemia, and pneumonitis, occurring in 27%, 13%, 6%, 2%, and 2% of patients, respectively. Other adverse events that were seen in greater than 10% of patients included decreased appetite, dyspnea, abdominal pain, vomiting, upper respiratory tract infection, decreased weight, rash, asthenia, night sweats, pneumonia, peripheral edema, and headache (Gopal et al., 2014).

In the phase III trial, more than 90% of patients experienced at least one adverse event. The most common adverse events in the idelalisib group were pyrexia, fatigue, nausea, chills, and diarrhea. Grade 3 or 4 neutropenia, thrombocytopenia, anemia, elevations in aminotransferases, and diarrhea occurred in 34%, 10%, 5%, 5%, and 4% of patients, respectively (Furman et al., 2014).

Adverse event profiles were fairly similar in both trials (Furman et al., 2014; Gopal et al., 2014). High incidences of hematologic laboratory abnormalities were seen in both trials. At least 55% of idelalisib-treated patients experienced neutropenia, ≥ 25% had anemia, and ≥ 17% had thrombocytopenia (Furman et al., 2014; Gopal et al., 2014). The manufacturers recommend that complete blood cell (CBC) counts be monitored in all patients at least every 2 weeks for the first 3 months. Refer to the prescribing information for recommendations on long-term monitoring (Gilead, 2014).

Severe adverse events and toxicities, including fatal or serious hepatotoxicity, diarrhea, or colitis were seen in both studies. This has prompted the Food and Drug Administration (FDA) to issue a Risk Evaluation and Mitigation Strategy (REMS) for idelalisib to make sure that practitioners are aware of the following black box warnings: fatal and/or serious hepatotoxicity, severe diarrhea, colitis, pneumonitis, and intestinal perforation. Aminotransferase monitoring should occur concurrently with CBC monitoring, as described above (Furman et al., 2014; Gopal et al., 2014; Gilead, 2014).

Of note, lymphocytosis has been observed with many agents targeting the BCR pathway, including idelalisib. This effect usually peaks around week 2 and resolves by week 12, as noted in the phase III trial. Lymphocytosis may be blunted, however, when idelalisib is used in combination with other B-cell–depleting agents such as rituximab (Furman et al., 2014).

## CONCLUSIONS

Idelalisib was granted accelerated approval on July 23, 2014, as monotherapy for the treatment of relapsed follicular B-cell NHL and SLL (Gilead press release, 2014). On the same day, it was also granted FDA approval for use in combination with rituximab for patients with CLL who would otherwise be candidates for rituximab monotherapy. Idelalisib is a viable option in patients with these indications, especially patients older than 65 or those with comorbidities such as moderate renal dysfunction (< 60 mL/min), poor bone marrow function or a CIRS ≥ 6. Idelalisib should also be considered in those CLL patients with poor prognostic features like unmutated IGHV or 17p deletions. Advanced practitioners should keep in mind, however, that the median duration of therapy in the larger, phase III trial was only 3.8 months and that long-term and late-onset toxicities have yet to be fully elucidated. Other potential combination regimens and indications for idelalisib are being evaluated in several phase III clinical trials (see Table 2). The results of these studies may expand and clarify idelalisib’s role and help to better incorporate it as an addition to standard therapies.

**Table 2:**
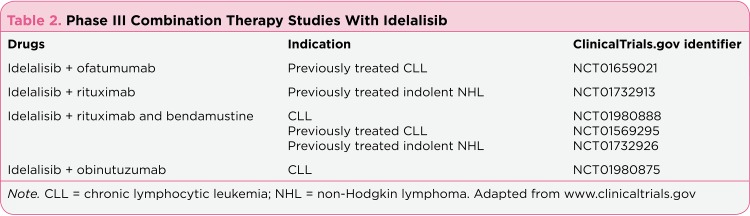
Phase III Combination Therapy Studies With Idelalisib

Idelalisib may be the first of several PI3K inhibitors to come. The pharmaceutical industry is actively pursuing PI3K inhibitors for many oncologic indications, including many solid tumor types, in light of the various PI3K isoforms and their roles in cell survival and development (Akinleye, Avvaru, Furqan, Song, & Liu, 2013). AMG-319, another selective PI3Kä inhibitor, is currently being tested in a phase I study for potential use in relapsed or refractory lymphoid malignancies (Brana & Siu, 2012). Copanlisib (BAY80-6946), a PI3Kα and β inhibitor, is being evaluated for use in indolent and aggressive NHL (Akinleye et al., 2013). in conclusion, the approval of idelalisib offers patients with CLL an option for therapy, as well as a possible therapeutic strategy for selected patients with FL and SLL.
